# Survival after aberrant left subclavian artery-esophageal fistula hemorrhage: A case report and technique review

**DOI:** 10.1016/j.jvscit.2025.101940

**Published:** 2025-08-05

**Authors:** Austin Clements, Kiara Touros, Greg Bearden, Vernon Horst

**Affiliations:** Department of Vascular Surgery, Baptish Health, Birmingham, AL

**Keywords:** Subclavian-esophageal fistula, Vascular anomaly, Hemorrhage, Gastrointestinal hemorrhage

## Abstract

Subclavian artery-esophageal fistula is a rare but potentially fatal vascular anomaly. Inherent to Downs syndrome, trisomy 21 presents with a variety of rare cardiac and vascular anomalies. Subclavian-esophageal fistulae are rare and often fatal complications of a right-sided aortic arch. We herein describe a rare case of a 31-year-old man with trisomy 21 who developed massive upper gastrointestinal hemorrhage secondary to an aberrant left subclavian artery-esophageal fistula requiring vascular surgical intervention. Although the etiology remains uncertain, we suspect that our patient's subclavian-artery esophageal fistula developed as a result of a combination of nasogastric tube compression and endotracheal/tracheostomy compression in the setting of aberrant left subclavian artery and a right-sided aortic arch. A broad differential and high index of suspicion is required in diagnosing a patient with trisomy 21 with known congenital cardiac and aortic arch anomaly presenting with acute upper gastrointestinal hemorrhage.

Aberrant great vessel anatomy consistently poses surgical challenges in diagnosis and management. The broad scope of such aberrant great vessel anatomy presents often as asymptomatic or incidental findings on imaging for other pathologies. However, timely diagnosis and treatment are essential in patient survival.^1^ Aortoesophageal fistulae commonly present with an aberrant right subclavian artery. However, rarely aortoesophageal fistulae presents with an aberrant retroesophageal left subclavian artery (ALSA).

In the case of our patient, an ALSA-esophageal fistula can present with dramatic gastrointestinal (GI) hemorrhage. Right-sided aortic arches and ALSA are formed as a result of the regression between the left common carotid artery and the left subclavian artery. ALSA usually takes its retroesophageal course, occasionally forming a Kommerell diverticulum and compressing the esophagus posteriorly.^2^ Esophageal compression combined with intraluminal injury or nasogastric tube placement has been associated with pressure necrosis and fistula formation.^3^ Currently, the literature cites the majority of arterial-esophageal hemorrhage secondary to foreign body or tracheostomy/endotracheal tube erosion.^3^, ^4^, ^5^

We report a patient with massive upper GI hemorrhage secondary to an ALSA-esophageal fistula after prolonged use of nasogastric tube and multiple esophagogastroduodenoscopies (EGDs). We also report our initial life-saving endovascular treatment technique and future operative planning strategy.

## Case report

We present a 31-year-old Caucasian man with a history of trisomy 21 and respiratory failure requiring endotracheal intubation after emergent strangulated umbilical hernia repair. He initially was admitted to our facility as a critical care transfer in acute respiratory failure for consideration of venovenous extracorporeal membrane oxygenation (ECMO) placement. Per institutional protocol and ECMO team evaluation, the patient was deemed not a suitable candidate for ECMO. Early in the patient's intensive care unit stay, a tracheostomy and percutaneous gastrostomy (PEG) tube were placed for long-term ventilation and enteral feeding. Approximately 1 month after tracheostomy and PEG tube placement, gastroenterology was reconsulted for evaluation of an upper GI hemorrhage and hematemesis. In retrospect, this would be identified as the sentinel hemorrhage. Emergent EGD was performed demonstrating retained blood within the stomach without an identifiable source of the hemorrhage. The patient was monitored closely in the intensive care unit on proton pump inhibitor therapy and transfused as needed. His hemoglobin during the sentinel hemorrhage was 6.8 g/dL and he received 1 U of packed red blood cells. His hemoglobin responded, increasing to 7.9 g/dL, which remained stable on multiple reevaluations.

**Fig 1:**
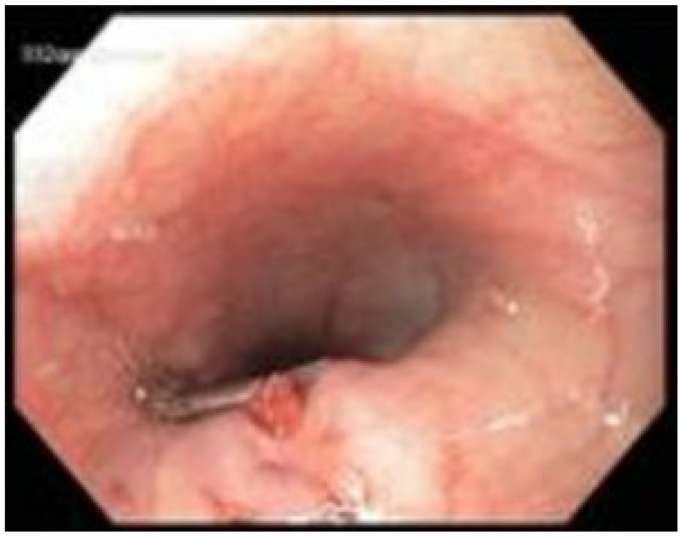
Posterior esophageal ulcer near upper esophageal sphincter with an endoclip and hemostasis.

Three days after the initial EGD, gastroenterology was reconsulted for recurrent upper GI hemorrhage. A second EGD was performed demonstrating a large volume of blood products retained within the esophagus and a pinpoint ulcerated arterial mucosal hemorrhage that was easily controlled with epinephrine injection and endoscopic hemoclip placement (Fig 1). The patient was ultimately transfused and stabilized. Given the identification of the GI hemorrhage source, computed tomography angiography (CTA) was foregone at this time.

**Fig 2:**
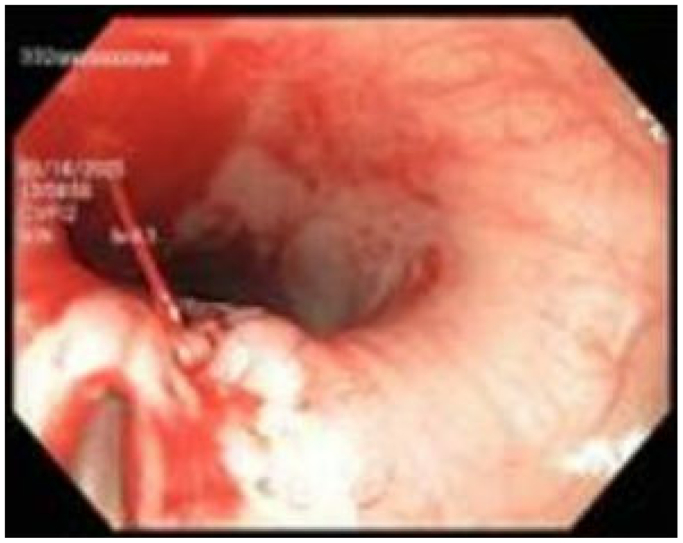
Hemorrhagic posterior esophageal ulcer near upper esophageal sphincter.

**Fig 3:**
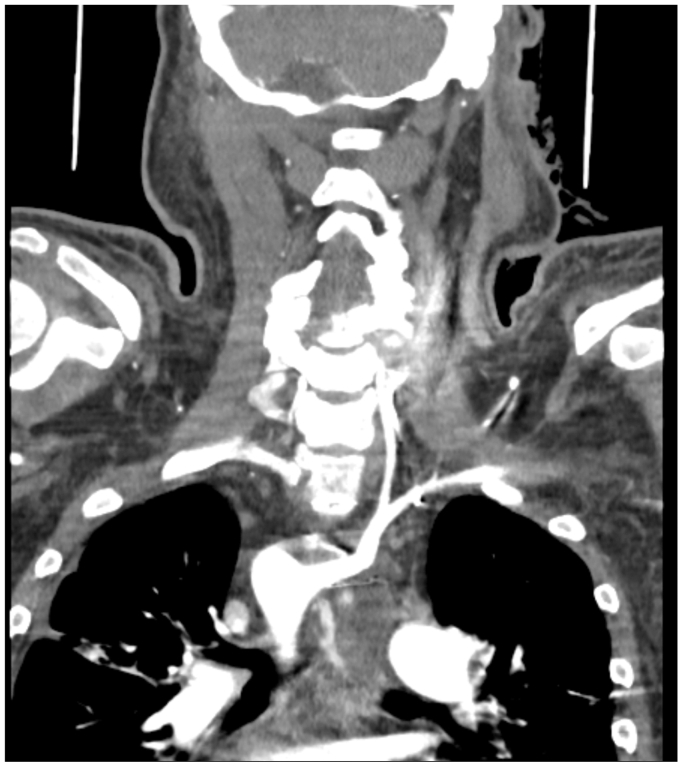
Coronal computed tomography angiography (CTA) showing the left subclavian artery-esophageal fistula.

**Fig 4:**
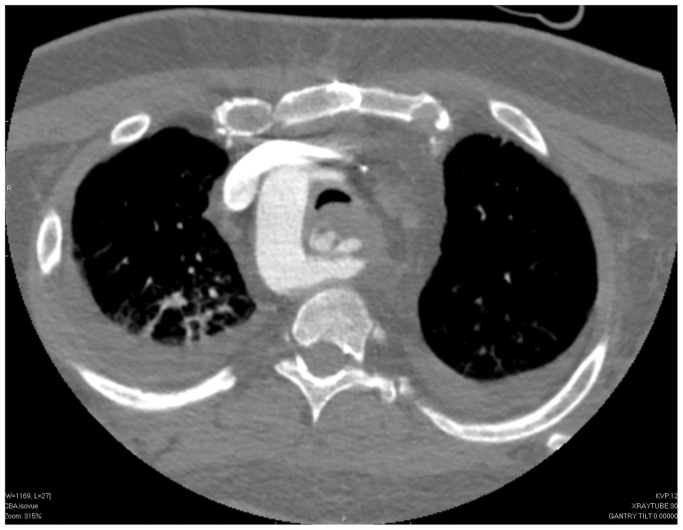
Axial computed tomography angiography (CTA) showing the left subclavian artery-esophageal fistula.

**Fig 5:**
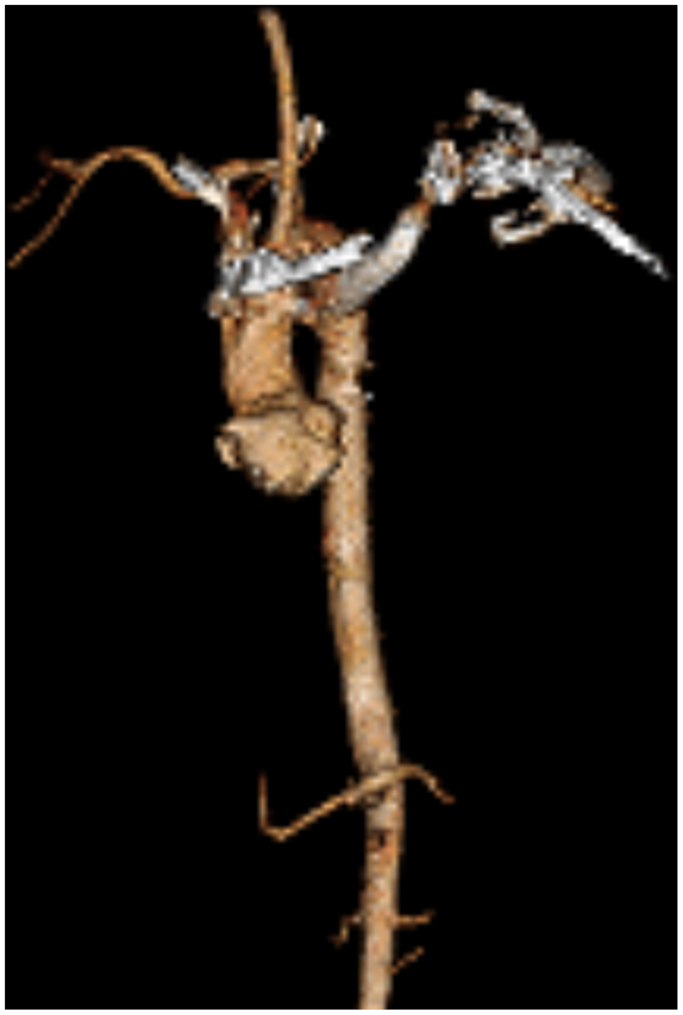
Computed tomography angiography (CTA) three-dimensional reconstruction showing the aberrant left subclavian artery/right sided aortic arch.

Two days after the second EGD, gastroenterology and ENT were consulted for massive upper GI hemorrhage and hemodynamic instability. The massive transfusion protocol along with vasopressors was initiated. Repeat EGD was performed demonstrating a large volume of blood within the upper GI tract and massive hemorrhage from the previously identified posterior esophageal lesion (Fig 2). This event would be the harbinger of the patient's future hospital course. EGD with epinephrine injection and endoscopic clip application was reattempted at that time without success. EGD demonstrated a large arterial hemorrhaging vessel within a cratered posterior esophageal ulcer (Fig 2). With assistance from gastroenterology, general surgery, thoracic surgery, interventional radiology, vascular surgery, cardiology, and ENT, the decision was made to tamponade the upper esophageal hemorrhage with epinephrine soaked gauze and proceed to the CT scanner for CTA to better delineate the source of the hemorrhage. CTA revealed a right-sided aortic arch, with an obvious aberrant left subclavian artery-esophageal fistula and active contrast extravasation (Fig 3, Fig 4, Fig 5). The patient was swiftly taken to the vascular suite for hemorrhage control while actively administering vasopressors and massive transfusion protocol.

**Fig 6:**
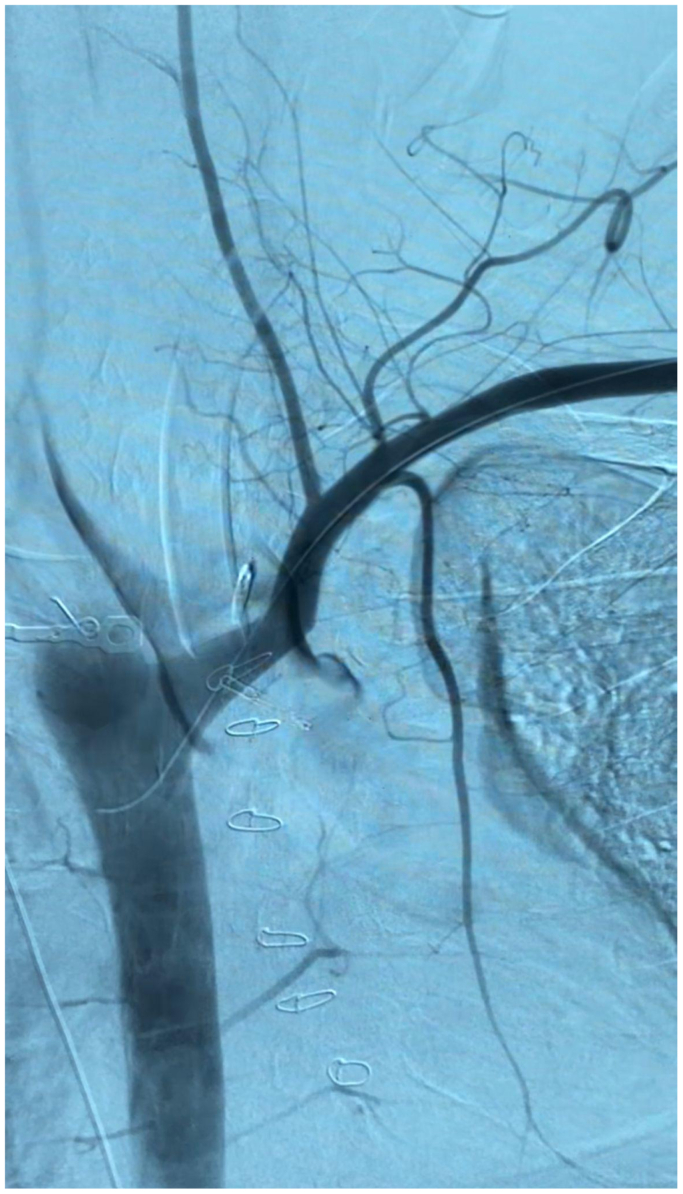
Left subclavian artery-esophageal fistula arteriogram with active extravasation.

Our operative strategy began with endovascular hemorrhage control with balloon tamponade and covered stent placement. Percutaneous access was obtained via the left brachial artery with a 7F sheath platform. Immediate pretreatment angiography was performed (Fig 6) demonstrating contrast extravasation from the left subclavian artery opacifying the esophageal lumen. An 0.035” angled glidewire system was advanced into the descending thoracic aorta. A 9 × 40 mm Boston Scientific (Marlborough, MA) Mustang balloon was advanced into position and inflated to profile for balloon tamponade. At this time, hemorrhage control had been obtained successfully and was verified by repeat CTA. Anesthesia was then able to continue to resuscitate the patient and prepare for definitive repair.

**Fig 7:**
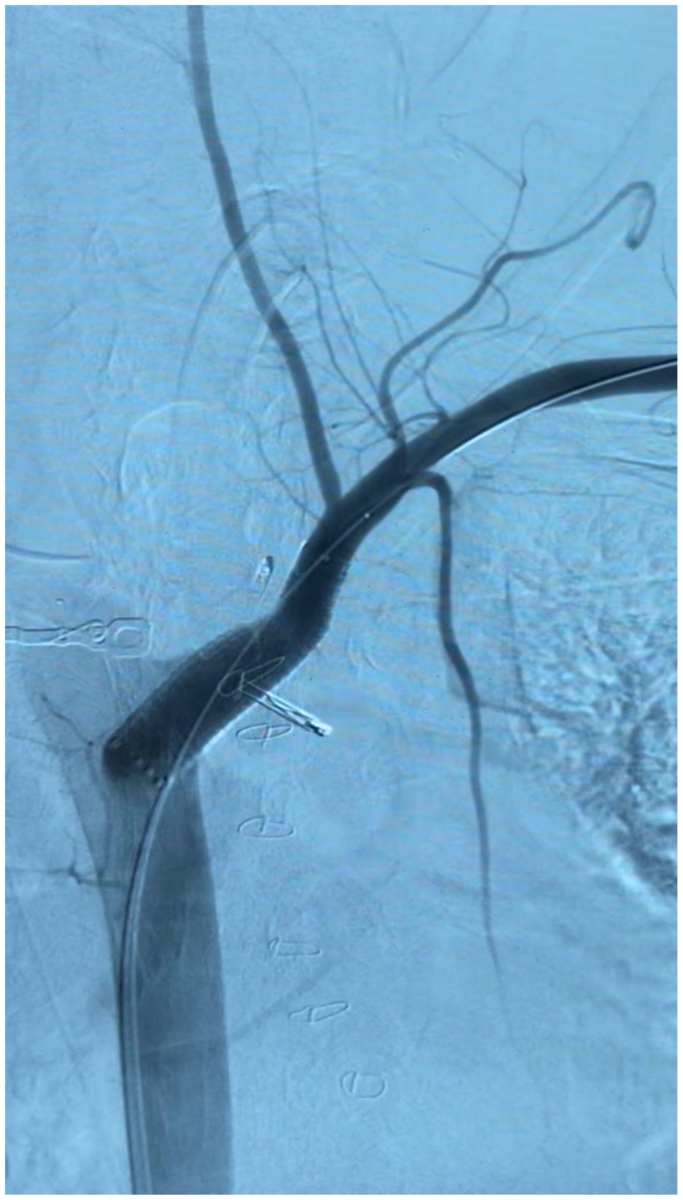
Left subclavian artery-esophageal fistula arteriogram, placement of 13 × 50 mm Gore Viabhan stent, without endoleak or extravasation.

After hemodynamics improved significantly, a covered endoprosthesis was placed. The 7F sheath platform was swiftly upsized to an 11F sheath platform. A 13 × 50 mm Gore Viabhan (W. L. Gore & Associates) stent was then advanced under fluoroscopic guidance into proper position, careful to land short of the left vertebral artery. Post-treatment angiography was performed and showed definitive resolution of left subclavian artery extravasation without endoleak (Fig 7). Exposure of the left brachial artery was then obtained. The 11F sheath platform was removed carefully under proximal and distal control. Given the size of sheath platform relative to the brachial artery diameter, autologous vein patch repair was performed with ipsilateral brachial vein harvest. After completion of the procedure, inline flow to the left hand was confirmed with Doppler signals at the brachial, radial, and ulnar arteries. The patient was then taken back to the intensive care unit in improving condition.

In the days after this procedure, the patient showed no further signs of GI hemorrhage and his respiratory status improved to minimal ventilatory support via tracheostomy. Multidisciplinary recommendations were made by the infectious diseases, vascular surgery, critical care, and gastroenterology departments. Eventually, his tracheostomy was decannulated and he was discharged successfully on long-term suppressive antibiotics (amoxicillin and clavulanate [Augmentin] 875 mg daily for 3 months) and dual antiplatelet therapy (aspirin 81 mg and clopidogrel 75 mg/d for 6 months) to a rehabilitation facility. Of note, future follow-up with our vascular surgery team has been established for thoracic endovascular aortic repair, left common carotid artery to left subclavian bypass, and proximal aberrant left subclavian artery ligation. Operative repair of the arterial-esophageal fistula will also be simultaneously undertaken by the thoracic surgery team. Owing to established distal enteral feeding access (PEG tube), containment of the fistula, and overall poor candidate for an open operation during the initial admission, the decision to repair the atrial-esophageal fistula was deferred until the patient could be optimized medically.

Overall, definitive comprehensive operative repair will be performed after the 3-month follow-up with repeat CTA imaging and multidisciplinary specialty discussions. Patient consent for publication was obtained per institution protocol.

## Discussion

An arterial-enteric fistula between a normal subclavian artery and the esophagus has largely been associated with the ingestion of foreign bodies. An aberrant retroesophageal ALSA elicits external compression posterior to the esophagus, thus increasing the susceptibility to pressure necrosis. In contrast with aberrant right subclavian artery, ALSA is a rarely reported congenital anomaly of the aortic arch.^5^

A subclavian artery-esophageal fistula must always be suspected in patients with known congenital vascular anomalies, chronic indwelling nasogastric tubes, endotracheal tubes, tracheostomies, foreign body ingestion, and presenting with massive hematemesis. Our case presentation follows the historical aortoesophageal syndrome first described by Chiari in 1914. He presented the Chiari triad: (1) sentinel hemorrhage followed by (2) hemostasis, ultimately resulting in (3) massive exsanguination and often death.^6^ Unique to our case, our swift endovascular treatment mitigated the finality of the Chiari triad. The placement of a Sengstaken-Blakemore tube has been touted as the only method to control hemorrhage temporarily, allowing for a more definitive diagnosis and treatment.^5^, ^6^, ^7^ However, in our case, we elected to pack off the upper esophagus with epinephrine-soaked gauze at the behest of our ENT colleagues. The patient was then able to be transported safely to the radiology department for CTA. Conversely, a Sengstaken-Blakmore or Minnesota tube would have been reasonable options for similar tamponade as well.

CTA remains essential for the accurate diagnosis of arterial-esophageal hemorrhage. Regardless of open or endovascular treatment options, without a definitive hemorrhage source, therapy is limited. Although not performed, CTA at the time of the patient's sentinel hemorrhage could have provided our vascular surgery team multiple approaches for repair, including open, endovascular, or hybrid approaches. Given the patient's hemodynamic instability, as well as the capabilities at our institution and vascular expertise, we elected to proceed initially with an endovascular approach.

Endovascular single-stage techniques have been described in controlling hemorrhage from both normal and aberrant left subclavian artery anatomies.^7^^,^^8^ We describe the emergent percutaneous left brachial artery approach and deployment of a Gore Viabahn stent (13 × 50 mm) within an 11F platform. Given the size of the sheath, the brachial artery did require vein patch angioplasty. The patient's survival and retained function of his left upper extremity speak to the success of our technique with minimal procedural complications. Despite requiring an open brachial artery repair, we elected for endovascular repair to avoid the well-studied open surgical repair that has been shown to carry extremely high mortality and morbidity rates.^4^^,^^5^^,^^8^ The risk of interim graft infection is paramount because our Gore Viabahn stent remains covering the arterial-esophageal fistula and is, by nature, presumed to be contaminated. As such, the patient was discharged on long-term suppressive antibiotics until definitive operative repair can be performed.

Currently, our future operative planning strategy begins with close 3-month follow-up and medical optimization for a thoracic endovascular aortic repair, left common carotid artery to left subclavian bypass, and proximal aberrant left subclavian artery ligation. Although open repair at the time of diagnosis provides better long-term outcomes in healthy asymptomatic patients, emergent open repair is associated with greater morbidity and mortality.^9^ Given the patient's hemodynamic instability, we elected to proceed with the life-saving endovascular deployment of a Gore Viabahn stent and plan for definitive repair should the patient survive the postoperative period.

In the end, the critical point is early recognition of an arterial-esophageal fistula in the Chiari triad with CTA and early intervention to prevent further hemodynamic demise. The physician's early recognition of ALSA depends on a high index of suspicion, critical care knowledge, and awareness of emergent endovascular and open surgical techniques.

## Conclusions

In patients with trisomy 21 with known vascular anomalies who require long-term multisystem critical care, an awareness of potential life-threatening hemorrhage sources must be maintained when presenting with an acute upper GI hemorrhage. We report the survival of a patients with Down syndrome with massive upper GI hemorrhage secondary to ALSA-esophageal fistula after a prolonged and complicated hospital course. Our report aims to add to the paucity of literature regarding the safety and efficacy of emergent endovascular repair of hemorrhagic ALSA-esophageal fistulae. Our report also stands as a careful reminder to vascular surgeons and critical care practitioners to maintain a broad differential in bleeding patients with congenitally aberrant vascular anatomy.

## Funding

None.

## Disclsoures

None.
